# Colon targeted drug delivery of branch-chained disulphide cross-linked polymers: design, synthesis, and characterisation studies

**DOI:** 10.1186/s13065-016-0226-4

**Published:** 2016-11-29

**Authors:** YongKhee Lau, Vuanghao Lim

**Affiliations:** Integrative Medicine Cluster, Advanced Medical and Dental Institute, Universiti Sains Malaysia, Bertam, 13200 Kepala Batas, Penang Malaysia

**Keywords:** Synthesis, Disulphide cross-linked polymer, Trithiol, Branch-chained, Colon drug delivery

## Abstract

Drug delivery directly to the colon is a very useful approach for treating localised colonic diseases such as inflammatory bowel disease, ulcerative colitis, and Crohn’s disease. The use of disulphide cross-linked polymers in colon targeted drug delivery systems has received much attention because these polymers are redox sensitive, and the disulphide bonds are only cleaved by the low redox potential environment in the colon. The goal of this study was to synthesise tricarballylic acid-based trithiol monomers for polymerisation into branch-chained disulphide polymers. The monomer was synthesised via the amide coupling reaction between tricarballylic acid and (triphenylmethyl) thioethylamine using two synthesis steps. The disulphide cross-linked polymers which were synthesised using the air oxidation method were completely reduced after 1 h of reduction with different thiol concentrations detected for the different disulphide polymers. In simulated gastric and intestinal conditions, all polymers had low thiol concentrations compared to the thiol concentrations in the simulated colon condition with *Bacteroides fragilis* present. Degradation was more pronounced in polymers with loose polymeric networks, as biodegradability relies on the swelling ability of polymers in an aqueous environment. Polymer P15 which has the loosest polymeric networks showed highest degradation.

**Electronic supplementary material:**

The online version of this article (doi:10.1186/s13065-016-0226-4) contains supplementary material, which is available to authorized users.

## Background

To date, oral drug delivery is the most preferred, common, convenient, and widely accepted route among the other routes available for drug administration [1]. The upper gastrointestinal (GI) tract is the major region for dissolution and absorption of orally administered drugs. Therefore, this approach is not suitable for delivery of drugs that are meant to be absorbed in the lower GI tract or for advanced biotechnology products, such as peptides and proteins, whereby undesirable side effects and treatment failure will occur. For this reason, researchers are focusing on developing techniques for targeting drugs to specific areas of the body, such as the lower GI tract. For example, colon specific drug delivery is a hot research topic [2–5], as such systems appear to be very useful for delivering drugs for localised treatment of colonic diseases such as inflammatory bowel disease, ulcerative colitis, and Crohn’s disease [6].

The role of colon specific drug delivery is not only limited for localised treatment but also crucial for systematic treatment [7]. Although colon specific drug delivery can also be achieved via rectal route, this route appeared to be less readily accepted and less appealing to patients. Moreover, study showed that it is difficult to deliver drugs to the proximal colon via the rectal route [8]. Lim et al. found that disulphide cross-linked polymers (as the drug carrier) were able to prevent premature drug release in the upper GI tract, thereby making colon drug targeting achievable [5]. The low redox potential environment of the human colon is the key to this system, as the disulphide bonds are cleaved only in this environment, thus releasing the drug only in the targeted location.

Disulphide cross-linked polymers synthesised by Lim et al. consists of one amide and one anhydride bond [5]. In this study, disulphide cross-linked polymers with 3 amide bonds were synthesised to reduce the solubility of the polymer due to the low solubility of amide bond. The idea of reducing the polymer solubility is to prevent premature disintegration of the polymer especially in stomach and small intestine. Recent studies have focused on using branch-chained disulphide polymers instead of linear-chained polymers because the former are less soluble; in contrast, linear-chained polymers are more soluble and easily degraded in low pH conditions [9]. In this study, branch-chained disulphide polymers based on tricarballylic acid were synthesised, and the polymers were characterised using various spectroscopic methods. Unlike previous study, the newly synthesised tricarballylic acid based disulphide polymers were investigated in simulated gastric, intestinal and colon condition. Successful synthesis of these polymers would provide potential carriers for use in colon specific drug delivery due to its abilities to remain intact in harsh gastric and intestinal condition, and disintegrate subsequently in low redox potential of colon environment.

## Experimental section

### Synthesis of monomers

#### Synthesis of (triphenylmethyl) thioethylamine (1)

2-aminoethane thiol (5.68 g, 50 mmol) and triphenylmethanol (13.02 g, 50 mmol) were stirred in trifluoroacetic acid (TFA) (50 mL) at room temperature for 3 h. The reaction was protected from moisture using a drying tube containing calcium chloride. The acid was evaporated off using a rotary evaporator to yield brown oil. The oil was triturated with diethyl ether to form a white precipitate that was filtered off and washed with diethyl ether. The white precipitate was partitioned between 1 mol L^−1^ NaOH and diethyl ether. The ether phase was evaporated off to yield a white solid **(1)**. Analytical calculations for C_21_H_21_NS: C 78.99%; H 6.58%; N 4.39%; S 10.03%. Analysis obtained: C 79.14%; H 7.11%; N 4.35%; S 10.01%. FT-IR (KBr disc): 3300 cm^−1^ (–NH stretch), 3052 cm^−1^ (–CH_2_–), 1950 cm^−1^ (benzene overtones), 930 cm^−1^ (–CH_2_– out-of-plane bands). ^1^H-NMR (400 MHz, Acetone-d_6_): δ7.3 (m, 15H, aromatic), δ2.9 (m, 2H, –C**H**_**2**_–NH–), δ2.6 (s, 2H, –N**H**_**2**_) and δ2.3 (m, 2H, –C**H**_**2**_–S–) (Additional file 1).

#### Synthesis of N,N′,N″-tris[2-(tritylsulfanyl)ethyl]propane-1,2,3-tricarboxamide (trityl monomer) (2)

**(1)** (6.72 g, 21 mmol) and tricarballylic acid (1.23 g, 7 mmol) were stirred in 100 mL of dichloromethane (DCM) for 10 min to ensure that the reactants were completely dissolved. 1-hydroxybenzotriazole hydrate (HOBt) (2.84 g, 21 mmol) was added to the mixture. The reaction flask was placed in an ice bucket to lower the reaction temperature to 0 °C. N-(3-dimethylaminopropyl)-N′-ethylcarbodiimide (EDC) (4.03 g. 21 mmol) was introduced into the reaction for amide coupling. The mixture was stirred for 8 h at 0 °C with a calcium chloride drying tube attached. Subsequently, the flask was stored at 0 °C for 18 h to allow complete reaction. The mixture was filtered to remove unwanted urea and washed with 5% citric acid, 2 mol L^−1^ sodium bicarbonate, and 2 mol L^−1^ sodium chloride. The mixture was dried using magnesium sulphate, and DCM was evaporated off using a rotary evaporator. The thin layer chromatography (TLC) revealed a dark black spot at R_f_ 0.67 when the solvent system of DCM: ethyl acetate (7:3) was used. The targeted spot was isolated using gravity column chromatography and a white coarse solid **(2)** was obtained. Analytical calculations for C_69_H_65_N_3_O_3_S_3_: C 76.63%; H 6.01%; N 3.89%; S 8.89%. Analysis obtained: C 76.45%; H 5.14%; N 3.51%; S 8.46%. FT-IR (KBr disc): 3281 cm^−1^ (–NH stretch), 3027 cm^−1^ (–CH_2_–), 1940 cm^−1^ (benzene overtones), 1642 cm^−1^ (–NHCO–), 743 cm^−1^ (–CH_2_– out-of-plane bands). ^1^H-NMR (400 MHz, CDCl_3_): δ7.25–7.4 (m, 45H, aromatic), δ6.0 (s, 3H, –N**H**–), δ2.85–3.0 (m, 7H, –C**H**_**2**_–S–, –C**H**–), δ2.25 (m, 10H, –C**H**_**2**_–NHCO–, –C**H**_**2**_–CONH–).

#### Synthesis of N,N′,N″-tris(2-sulfanylethyl)propane-1,2,3-tricarboxamide (trithiol monomer) (3)

**(2)** (5.4 g, 5 mmol) was dissolved in DCM. The mixture was treated with 6 mL of TFA followed by 1 mL of triethylsilane (TES). The mixture was stirred for 3 h at room temperature. The solvent was evaporated off and the compound was washed with diethyl ether to produce a white powdery solid **(3)**. Analytical calculations for C_12_H_23_N_3_O_3_S_3_: C 40.73%; H 6.51%; N 11.88%; S 27.16%. Analysis obtained: C 41.22%; H 6.83%; N 11.52%; S 25.89%. FT-IR (KBr disc): 3285 cm^−1^ (–NH stretch), 2550 cm^−1^ (–SH), 1638 cm^−1^ (–NHCO–). ^1^H-NMR (400 MHz, CDCl_3_): δ6.7 (s, 3H, –N**H**–), δ3.1–3.4 (m, 7H, –CH–, –C–**H**_**2**_–SH), δ2.4–2.6 (m, 10H, C**H**_**2**_NHCO, C**H**_**2**_CONH).

### Oxidative polymerisation of (3)

**(3)** was placed in ammonium bicarbonate buffer (0.1 mol L^−1^, pH 8.3), and the mixture was stirred to ensure complete dissolution. Dimethyl sulphoxide (DMSO) was later added until approximately 50% of the solids were dissolved. The mixture was stirred continuously and exposed to open air for 24–48 h [10]. The reaction was terminated when no more thiol could be detected using sodium nitroprusside reagent. The resultant white suspension was filtered and washed with water and methanol to produce a powdery white solid. Different molar ratios between the trithiol monomer and 2,2′-(ethylenedioxy)diethanethiol (dithiol monomer) were employed as described below to obtain different polymers:

Polymer P10—trithiol monomer only

Polymer P11—1.0 trithiol monomer: 1.0 dithiol monomer

Polymer P12—1.0 trithiol monomer: 2.0 dithiol monomer

Polymer P15—1.0 trithiol monomer: 5.0 dithiol monomer

Polymer P21—2.0 trithiol monomer: 1.0 dithiol monomer

Polymer P51—5.0 trithiol monomer: 1.0 dithiol monomer

The polymers then were subjected to the analyses described below.

### Fourier transform infrared spectroscopy (FT-IR)

FT-IR spectra using KBr discs were generated using a Nexus FT-IR spectrophotometer (Thermo Nicolet, Madison, USA).

### Proton nuclear magnetic resonance spectroscopy (^1^H-NMR)

^1^H-NMR spectra were recorded in acetone-d_6_ and Deuterated Chloroform (CDCl_3_) on a Bruker AC 400 at 400 MHz (Stuttgart, Germany), and all deuterated solvents for NMR were obtained from Sigma Chemical (St. Louis, USA).

### Elemental analysis (CHNS) and melting point tests

The elemental analysis was conducted by combustion analysis using a CHNS/O analyser (Perkin-Elmer 2400, MA, USA); combustion temperature was 950 °C and reduction occurred at 550 °C. All melting points were measured with a melting point apparatus (Gallenkamp, London, England).

### Raman spectroscopy

Raman spectra were recorded using a Jobin–Yvon HR 800 UV Raman spectrometer (Lower Hutt, New Zealand). The incident laser excitation wavelength was 514.5 nm, with output of 20 mW, and the spectra were recorded from 100 to 3000 cm^−1^.

### Scanning electron microscope-energy dispersive X-ray (SEM-EDX)

A sample of each polymer was sputtered with gold using a Polaran (Fisons Instruments, Uckfield, UK) SC 515 sputter coater. Pictures were taken with a SEM LEO Stereoscan 4201 microscope (Leica Electron Optics, Cambridge Instruments Ltd, Cambridge, UK) with up to 1000× magnification. The EDX analysis was performed using the detection-microanalysis-system INCA 400 (Oxford Instruments PLC, Bucks, UK) using electron beam spot sizes <50 nm.

### Solubility test for disulphide cross-linked polymers

Various types of organic solvents such as DCM, DMSO, chloroform, acetone, acetonitrile, ethanol, water and phosphate buffer pH 1.2, 6.8 and 7.4 were used for the solubility test. 3 mg of polymer P10 was inserted into an eppendorf tube. 1 mL of DCM was added into the tube. The cap of the tube was closed and the mixture was spinned for 5 min using homogeniser. The mixture was observed under bright light to determine the solubility of the polymer. The steps were repeated for different organic solvents and phosphate buffers with different polymers.

### Chemical reduction studies of disulphide cross-linked polymers

For each type of disulphide cross-linked polymer, a 0.3 g sample and acetic acid (1.3 mL) were dissolved in 10 mL of distilled water in a 3-neck round bottom flask. The mixture was purged with oxygen-free nitrogen for 15 min. The mixture was refluxed at 100 °C, and zinc dust (1.95 g, 30 mmol L^−1^) was then added slowly into the flask while stirring [11]. Using an high performance liquid chromatography (HPLC) microsyringe, 10 µL of sample was withdrawn from the side arm of the flask and diluted with Sørensen’s phosphate buffer (pH 7.4) containing 0.006 mol L^−1^ Ethylenediaminetetraacetic acid (EDTA). The diluted sample was mixed well and filtered through a Pasteur pipette with pre-inserted cotton wool. Finally, 1 mL of the sample solution was used to measure the thiol content.

### Assay for thiol using Ellman’s reagent and the Beer-Lambert equation

To measure the thiol content of a sample, 0.1 mol L^−1^ of Ellman’s reagent was prepared in Sørensen’s phosphate buffer pH 7.4. A set of sample tubes, each containing 50 µL of Ellman’s reagent and 2.5 mL of Sørensen’s phosphate buffer (pH 7.4 or 8.0), was prepared. To each sample tube, 250 µL of each standard or the polymers were added; 250 µL of Sørensen’s phosphate buffer were added to the blank (reference) cuvette instead of thiol-containing solution. The tubes were mixed and left stirring for 15 min at room temperature to enable the thiol exchange to occur. The ultraviolet (UV) absorbance then was measured at 412 nm using a 1 cm cell. The Beer-Lambert equation was applied to calculate the thiol concentration in each sample:

$${\text{C}} = {\text{A}}/ {{\upvarepsilon }} \cdot {\text{d}}$$

where C is the thiol concentration (mol L^−1^), A is absorbance, d is cell path length (1 cm), and ε is the molar absorption coefficient in Sørensen’s phosphate buffer pH 7.4 (14,150 L mol^−1^ cm^−1^).

### In vitro dissolution studies

#### Degradation in simulated gastric fluid

In order to prepare simulated gastric fluid, 2 g of sodium chloride (NaCl) and 3.2 g of pepsin powder were dissolved in 0.1 mol L^−1^ hydrochloric acid [12]. For this assay, 1000 mL of simulated gastric fluid were placed in the vessel of the USP-standard dissolution apparatus (Agilent Technologies, Santa Clara, USA). The fluid was allowed to equilibrate to a temperature of 37 ± 0.5 °C. A Visking dialysis tube containing 0.4 g of polymer was subjected to the fluid for 2 h with the stirring speed set at 50 rpm. To evaluate the degradation of disulphide polymers, 1 mL samples were taken at pre-set time intervals (2, 5, 7, 10, 15, 20, 30, 40, 50, 60, 70, 80, 100 and 120 min). For every 1 mL of sample taken, 1 mL of simulated gastric fluid was added to the reaction mixture. Experiments were repeated 3 times for each disulphide polymers.

#### Degradation in simulated intestinal fluid

Simulated intestinal fluid were prepared by mixing 77 mL of 0.2 mol L^−1^ sodium hydroxide with 250 mL solution containing 6.8 g of KH_2_PO_4_. The resulting mixture was mixed with 500 mL of distilled water. 10 g of pancreas powder was added and stirred until the powder was completely dissolved. The final mixture was diluted to a final volume of 1000 mL by addition of distilled water [12]. After the previous experiment was concluded, 1000 mL of simulated intestinal fluid were placed in a new vessel, and the fluid was allowed to equilibrate to a temperature of 37 ± 0.5 °C. The Visking dialysis tube containing polymer from “Degradation in simulated gastric fluid” section was recovered and placed in the vessel containing simulated intestinal fluid. Further degradation tests were conducted for 3 h with the stirring speed set at 50 rpm. To evaluate the degradation of disulphide polymers, 1 mL of sample was removed at pre-set time intervals (5, 10, 20, 40, 60, 80,100, 120, 140 and 180 min). For every 1 mL of sample taken, 1 mL of simulated intestinal fluid was added to the reaction mixture. Experiments were repeated 3 times for each disulphide polymers.

#### Degradation in simulated colon conditions

The Visking dialysis tube containing polymer from “Degradation in simulated intestinal fluid” section was opened, and a *Bacteroides fragilis* pellet pre-separated from bacterial culture was added together with 15 mL of Sørensen’s phosphate buffer pH 7.4. A closed sac was formed by tying a knot at the open end of the tube. The sac was placed in a 100 mL conical flask (incubation vessel) containing 90 mL of Sørensen’s phosphate buffer. The mouth of the conical flask was covered and sealed with rubber bung and flushed with oxygen-free nitrogen via a sterile needle. The incubation was continued in a shaking water bath at 37 °C with continuous purging of oxygen-free nitrogen. Samples were collected according pre-set duration time intervals of incubation (0.5, 1, 1.5, 2, 2.5, 3, 4, 5, 6, 7, 8, 10, 16, 20, 24, 30, 40, 50, 60 and 70 h). Experiments were repeated 3 times for each disulphide polymers.

#### Control incubations

Experimental controls for degradation in simulated colon conditions were conducted in two sets, comprising of the disulphide cross-linked polymer incubated in Sørensen’s phosphate buffer alone without presence of bacteria and incubation of *B. fragilis* suspension in buffer alone without the polymer.

#### Determination of thiol concentration

The method described in section assay of thiol was used for the determination of thiol concentration.

### Statistical analysis

The final thiol concentrations at hour 2 of the simulated gastric condition, hour 3 of the simulated intestine condition, and hour 70 of the simulated colon condition for the different polymers were analysed using one-way analysis of variance (ANOVA) (IBM SPSS Statistics Version 20). Post-hoc analysis using Dunnett’s (2-sided) test was conducted when a statistically significant difference at p < 0.05 was obtained. The final thiol concentrations at hour 70 (polymer + bacteria, polymer only and bacteria only) for different polymers in simulated colon condition were also analysed using one-way ANOVA. Post-hoc analysis using Dunnett’s (2-sided) test was conducted and a statistically significant difference at p < 0.05 was obtained.

## Results and discussion

### Synthetic route

**Fig. 1:**
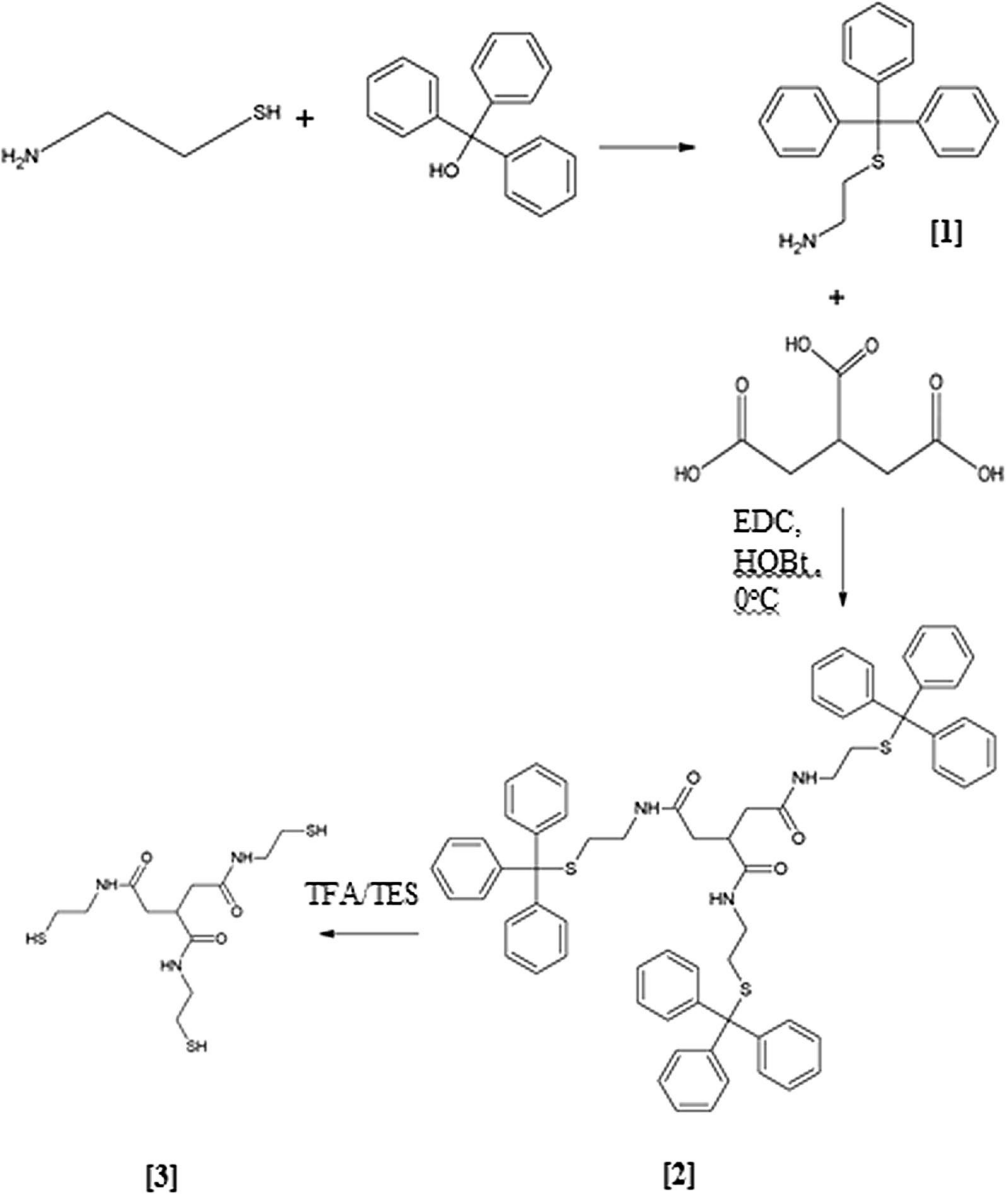
Synthetic routes for preparing *N*,*N*′,*N*″-tris(2-sulfanylethyl)propane-1,2,3-tricarboxamide **(3)**

The synthetic route used to create trithiol monomer **(3)** is demonstrated in Fig. 1. **(1)** was obtained in bulk following the protection reaction with triphenylmethanol. The amide coupling reaction of **(1)** with tricarballylic acid gave a low yield of **(2)**. **(3)** was obtained in high yield via the deprotection reaction to remove trityl protecting groups.

### Elucidation of (1)

**(1)** was obtained as a white powdery solid (14.3 g) with percentage yield of 88–90%. The melting point was recorded at 94–96 °C. TLC analysis of the compound revealed a dark black spot at R_f_ 0.7 when the solvent system contained ethyl acetate: methanol: acetic acid (6:3:1) (v/v/v). The spot turned violet in colour after being sprayed with ninhydrin reagent, which showed the presence of amine group [13]. The peaks at 3309–3371 cm^−1^ indicated the presence of amine groups, and those at 1700–1953 cm^−1^ showed the presence of aromatic groups. Triphenylmethyl protecting groups were shown to have successfully attached to thiol with free amine in the structure. The result was further confirmed by ^1^H-NMR analysis, which showed the presence of triphenylmethyl groups as multiplets at δ7.0–7.3 ppm. Elemental analysis revealed a similar percentage of elements calculated from the empirical formula of the structure (C_21_H_21_NS).

### Elucidation of (2)

**(2)** was a white coarse solid (1.76 g) with percentage yield of 20–25% and a melting point of 216–218 °C. Dichloromethane: ethyl acetate (7:3) (v/v) was the solvent system used for TLC analysis, and a dark black spot was observed at R_f_ 0.65. The peaks at 3281 cm^−1^ and 1642 cm^−1^ showed the presence of amide and carbonyl groups, respectively. Aromatic protecting groups were present at peaks 1773–1949 cm^−1^. These results showed that amide coupling between **(1)** and tricarballylic acid had occurred. ^1^H-NMR analysis showed the presence of aromatic protecting groups as multiplets at δ7.1–7.4 ppm, which supported the presence of amide groups. Elemental analysis of **(2)** revealed a similar percentage of elements calculated from the empirical formula of the structure (C_69_H_65_N_3_O_3_S_3_).

### Elucidation of (3)

Deprotection of **(2)** yielded a grey powdery solid (1.33 g) with percentage yield of 70–80% and a melting point of 195–197 °C. TLC analysis of the compound showed the absence of a dark spot under short ultraviolet wavelength (254 nm), indicating the absence of conjugated bonds after the successful removal of the trityl protecting group. A new peak was detected at 2550 cm^−1^, indicating the presence of a thiol group, and a peak at 1638 cm^−1^ showed the presence of the carbonyl group of amide. The overtone peaks of benzene in the 1700–1900 cm^−1^ region were absent, which illustrated that the aromatic protecting groups were successfully removed and the resulting compound **(3)** contained free thiols. These result were supported by the absence of region δ7–7.5 ppm and the emergence of the SH peak at 2553 cm^−1^ in ^1^H-NMR and Raman spectrometry, respectively. Elemental analysis of **(3)** revealed a similar percentage of elements calculated from the empirical formula of the structure (C_12_H_23_N_3_O_3_S_3_).

### Physical characterisation of disulphide cross-linked polymers

#### Solubility test for disulphide cross-linked polymers

Various types of organic solvents, such as DCM, DMSO, chloroform, acetone, acetonitrile, ethanol, water and phosphate buffer pH 1.2, pH 6.8 and pH 7.4 were used for the solubility test (Table 1). It was found that all polymers are insoluble in DCM, chloroform, acetone, acetonitrile, ethanol, water and phosphate buffers. Polymer P15 and polymer P12 were found to be soluble and partially soluble in DMSO, respectively. An increase in the molar ratio of dithiol led to increased polymer solubility in DMSO. Thus, DMSO was chosen as the oxidative agent because of its essential role as a solvent to effect dissolution of the trithiol monomer. Use of DMSO significantly increased the effectiveness of the entire polymerisation process. DMSO has been found to be useful as a mild oxidising agent, especially for simple organic thiols [14].

**Table 1:** Results of the solubility test of the synthesised polymers at different molar ratios with various solvents and pHs

Polymer/solvents	Solubility test					
	P10	P11	P12	P15	P21	P51
DCM	Insoluble	Insoluble	Insoluble	Insoluble	Insoluble	Insoluble
DMSO	Insoluble	Insoluble	Partially soluble	Soluble	Insoluble	Insoluble
Chloroform	Insoluble	Insoluble	Insoluble	Insoluble	Insoluble	Insoluble
Acetone	Insoluble	Insoluble	Insoluble	Insoluble	Insoluble	Insoluble
Acetonitrile	Insoluble	Insoluble	Insoluble	Insoluble	Insoluble	Insoluble
Ethanol	Insoluble	Insoluble	Insoluble	Insoluble	Insoluble	Insoluble
Water	Insoluble	Insoluble	Insoluble	Insoluble	Insoluble	Insoluble
pH 1.2	Insoluble	Insoluble	Insoluble	Insoluble	Insoluble	Insoluble
pH 6.8	Insoluble	Insoluble	Insoluble	Insoluble	Insoluble	Insoluble
pH 7.4	Insoluble	Insoluble	Insoluble	Insoluble	Insoluble	Insoluble

#### Physical appearance of disulphide cross-linked polymers

Table 2 describes the physical appearance of the synthesised disulphide cross-linked polymers of different molar ratios.

**Table 2:** Physical appearance of synthesised disulphide polymers

Polymer	Physical appearance
P10	Rugged white solid
P11	White solid
P12	White solid
P15	Slightly sticky white solid
P21	Powdery white solid
P51	Rugged white solid

### FT-IR analysis of disulphide cross-linked polymers

FT-IR results for the disulphide cross-linked polymers are shown below:

Polymer P10: FT-IR (KBr disc) = 3289 cm^−1^ (–NH stretch), 2913 cm^−1^ (–CH_2_–), 1639 cm^−1^ (–NHCO–).

Polymer P11: FT-IR (KBr disc) = 3297 cm^−1^ (–NH stretch), 2913 cm^−1^ (–CH_2_–), 1642 cm^−1^ (–NHCO–), 1103 cm^−1^ (C–O–C stretch).

Polymer P12: FT-IR (KBr disc) = 3289 cm^−1^ (–NH stretch), 2913 cm^−1^ (-CH_2_-), 1642 cm^−1^ (–NHCO–), 1103 cm^−1^ (C–O–C stretch).

Polymer P15: FT-IR (KBr disc) = 3285 cm^−1^ (–NH stretch), 2905 cm^−1^ (–CH_2_–), 1642 cm^−1^ (–NHCO–), 1107 cm^−1^ (C–O–C stretch).

Polymer P21: FT-IR (KBr disc) = 3285 cm^−1^ (–NH stretch), 2913 cm^−1^ (–CH_2_–), 1642 cm^−1^ (–NHCO–), 1099 cm^−1^ (C–O–C stretch).

Polymer P51: FT-IR (KBr disc) = 3285 cm^−1^ (–NH stretch), 2913 cm^−1^ (–CH_2_–), 1638 cm^−1^ (–NHCO–), 1095 cm^−1^ (C–O–C stretch).

For all six polymers, FT-IR results showed the disappearance of the sulfhydryl peak at 2550 cm^−1^, indicating that the polymerisation of thiol monomers into disulphide polymers was successful. Peaks were detected at 3289 and 1642 cm^−1^, showing the existence of amide groups in the polymers. A new peak of 1103 cm^−1^ was detected for all polymers except polymer P10, which indicated the presence of C–O–C stretch of the dithiol monomers, which further confirmed that the disulphide polymer was successfully synthesised. The C–O–C peak was not observed in polymer P10 because this polymer was polymerised solely from trithiol monomers. The intensity of the C–O–C peak increased as the feed molar ratio of the dithiol monomer used increased. From the FT-IR results, polymers P15 and P51 showed the highest and lowest intensity for the C–O–C peak, respectively.

### SEM-EDX micrographs

**Fig. 2:**
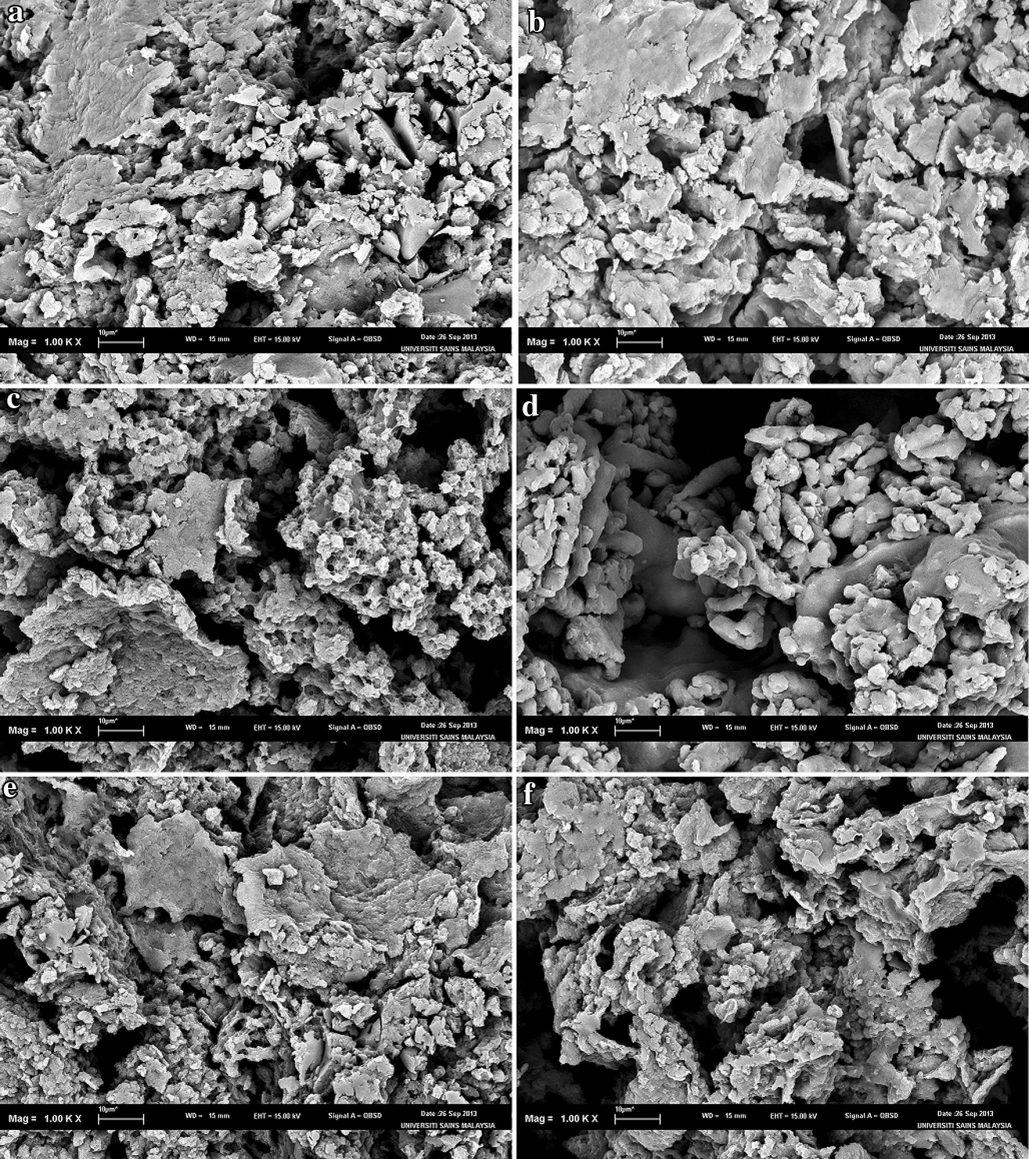
Scanning electron micrographs at ×1000 magnification of polymers **a** P10, **b** P11, **c** P12, **d** P15, **e** P21, and **f** P51

SEM was used to examine the surface morphology of the synthesised disulphide cross-linked polymers. SEM is routinely used to generate high-resolution images of shapes of objects and to show spatial variations in chemical compositions. The distribution of elements can be detected using EDX. The SEM images showed that the surfaces of all six disulphide polymers were rough and uneven (Fig. 2).

SEM images for polymer P10 with magnification up to 1000× revealed a coarse and rough surface. Polymerisation of only the trithiol monomer contributed to the more compact zone within the polymer network, ultimately leading to the formation of the rough surface morphology [5]. The surface of polymers composed of trithiol/dithiol monomers appeared to be more porous compared to the polymers composed solely of trithiol monomer. The degree of porosity increased when the molar ratios of dithiol monomers increased. Polymer P15 had the most porous surface among all of the polymers due to the high proportion of dithiol monomer, which led to the formation of a loose polymer network. The surface morphology of polymer P12 was less porous than that of P15 but more porous than that of P11, P21, P51, and P10. Several studies reported that the tighter polymers have a more rugged surface [5, 15], which is in agreement with the SEM results.

**Fig. 3:**
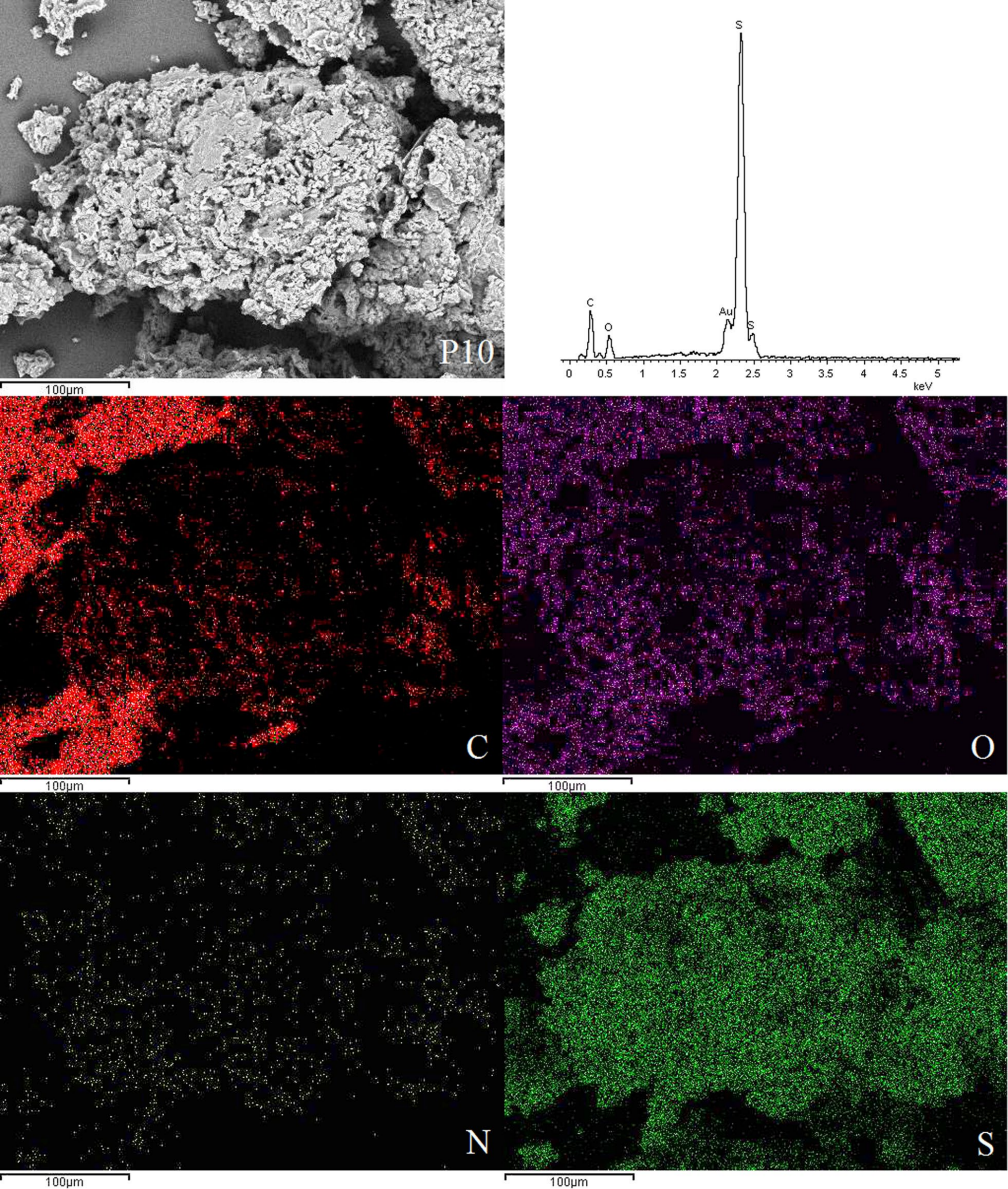
SEM micrographs at ×300, EDX and elemental maps for carbon (C), oxygen (O), sulphur (S), and nitrogen (N) for the same region for polymers P10

**Fig. 4:**
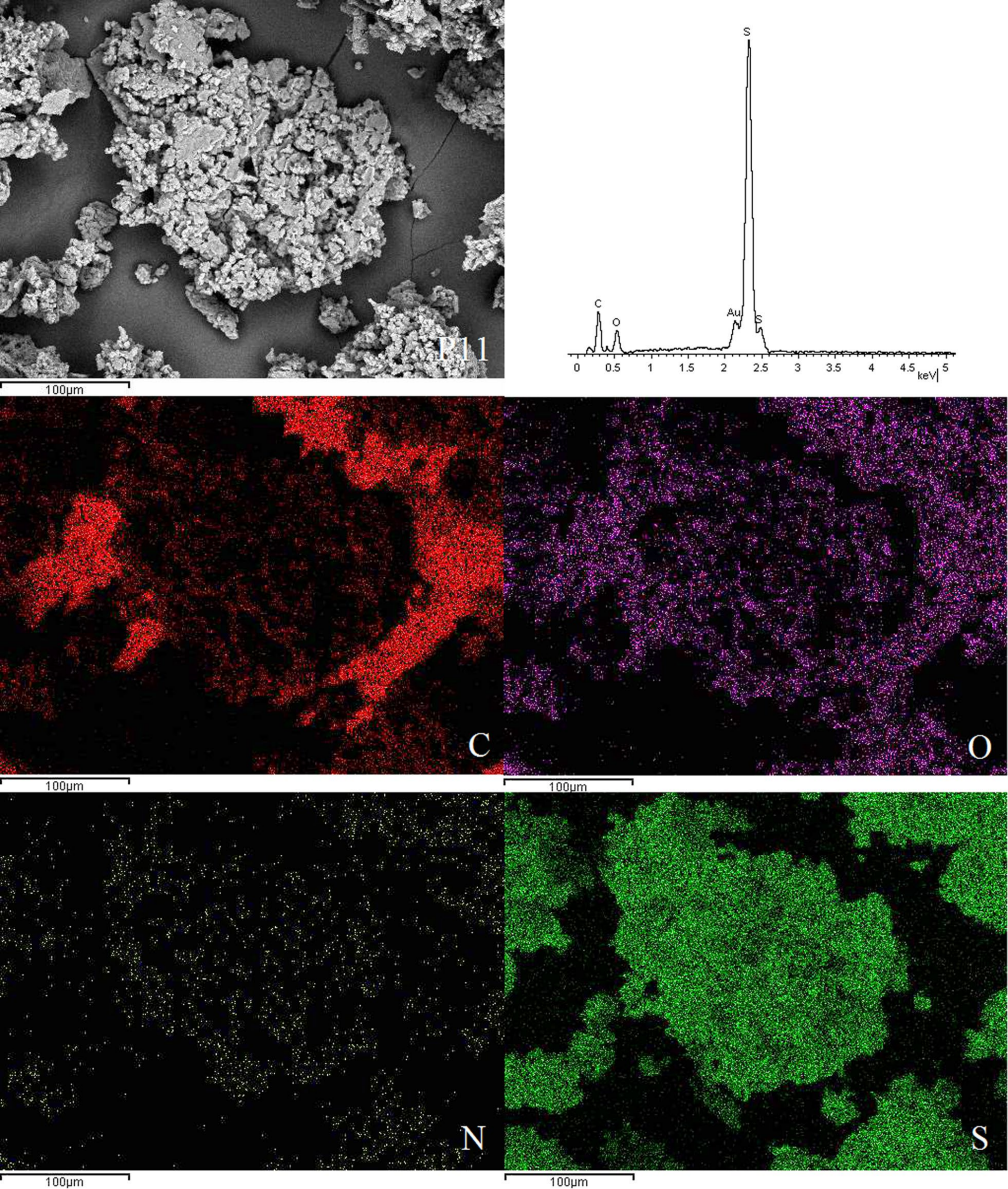
SEM micrographs at ×300, EDX and elemental maps for carbon (C), oxygen (O), sulphur (S), and nitrogen (N) for the same region for polymers P11

**Fig. 5:**
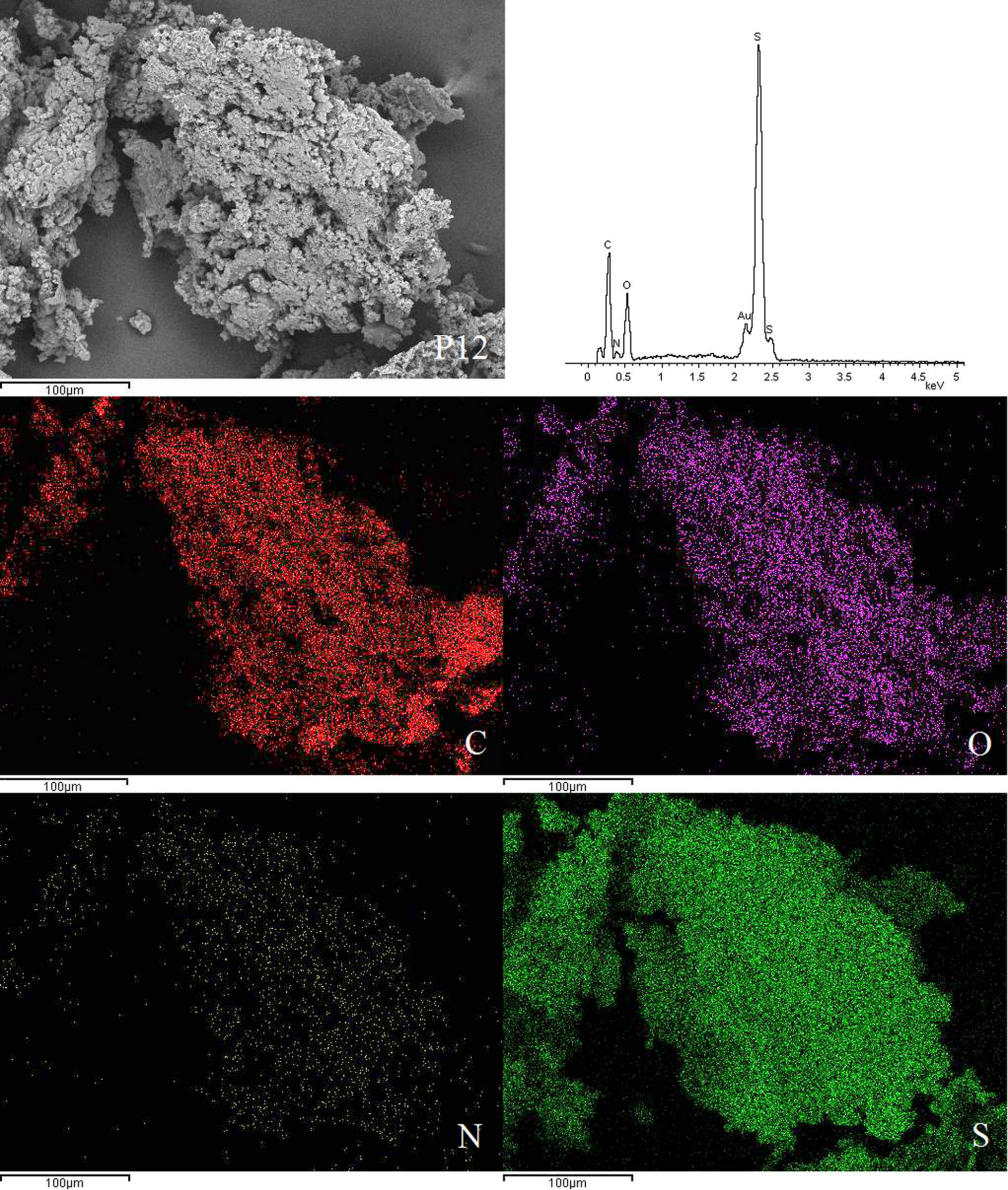
SEM micrographs at ×300, EDX and elemental maps for carbon (C), oxygen (O), sulphur (S), and nitrogen (N) for the same region for polymers P12

**Fig. 6:**
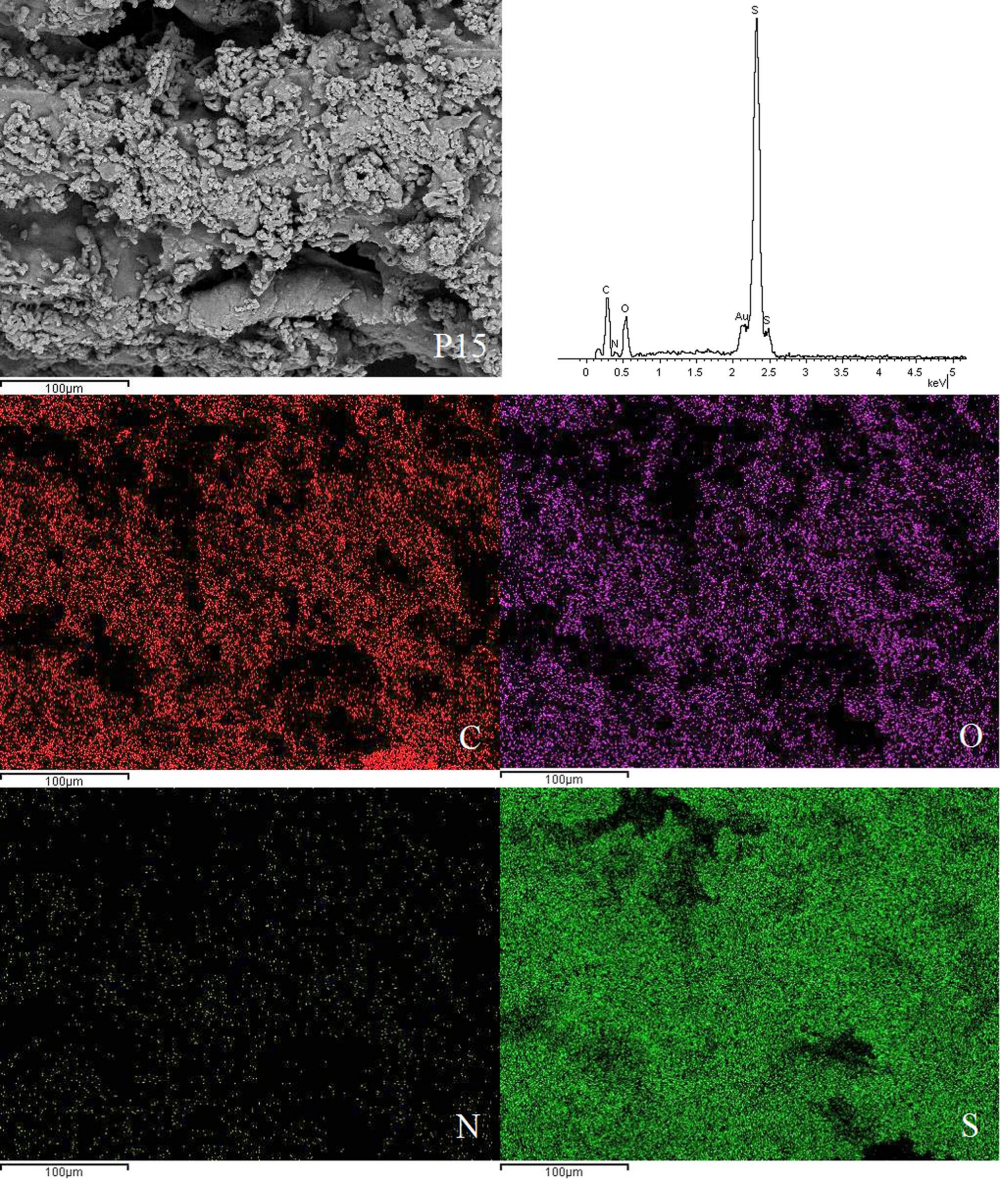
SEM micrographs at ×300, EDX and elemental maps for carbon (C), oxygen (O), sulphur (S), and nitrogen (N) for the same region for polymers P15

**Fig. 7:**
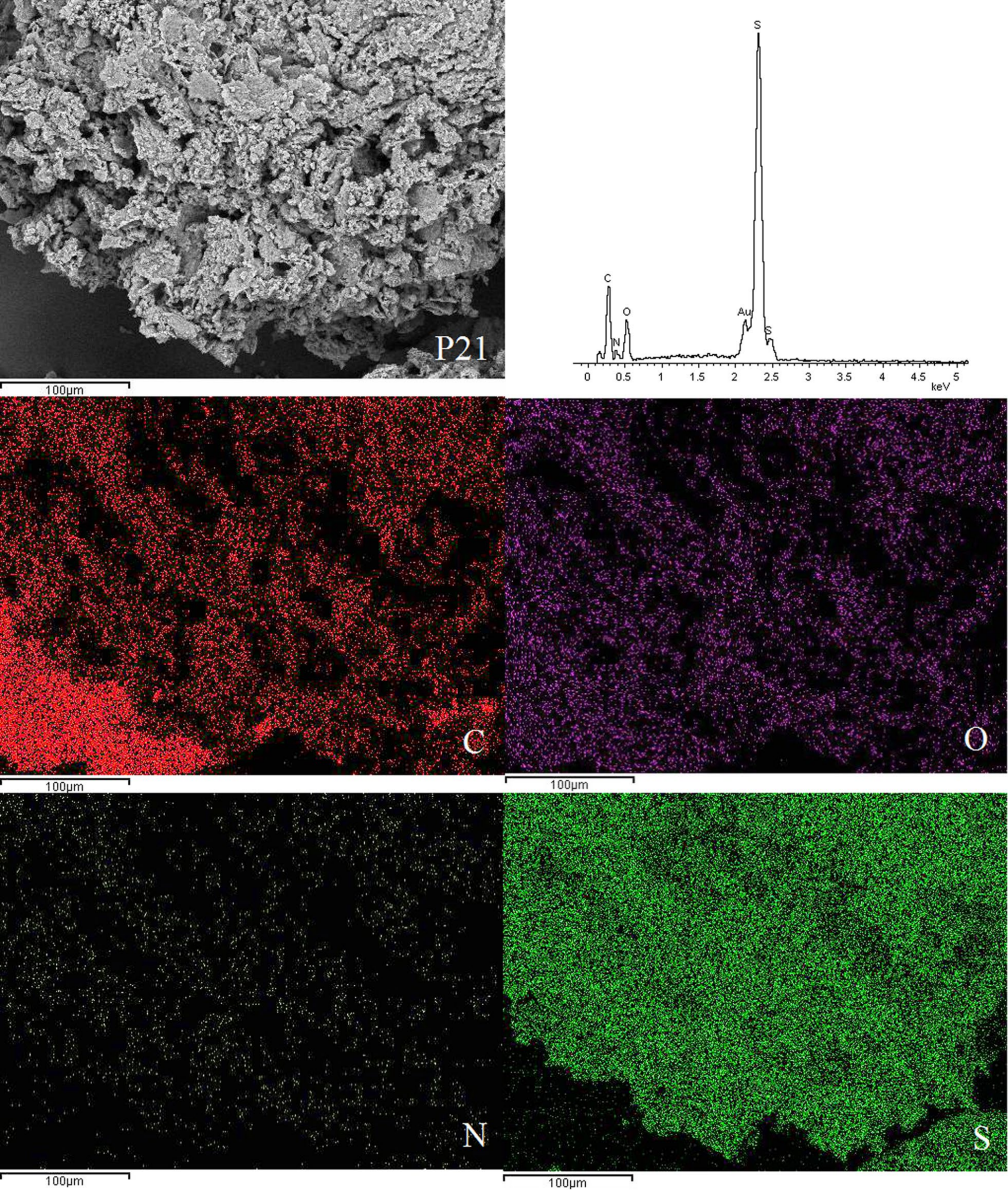
SEM micrographs at ×300, EDX and elemental maps for carbon (C), oxygen (O), sulphur (S), and nitrogen (N) for the same region for polymers P21

**Fig. 8:**
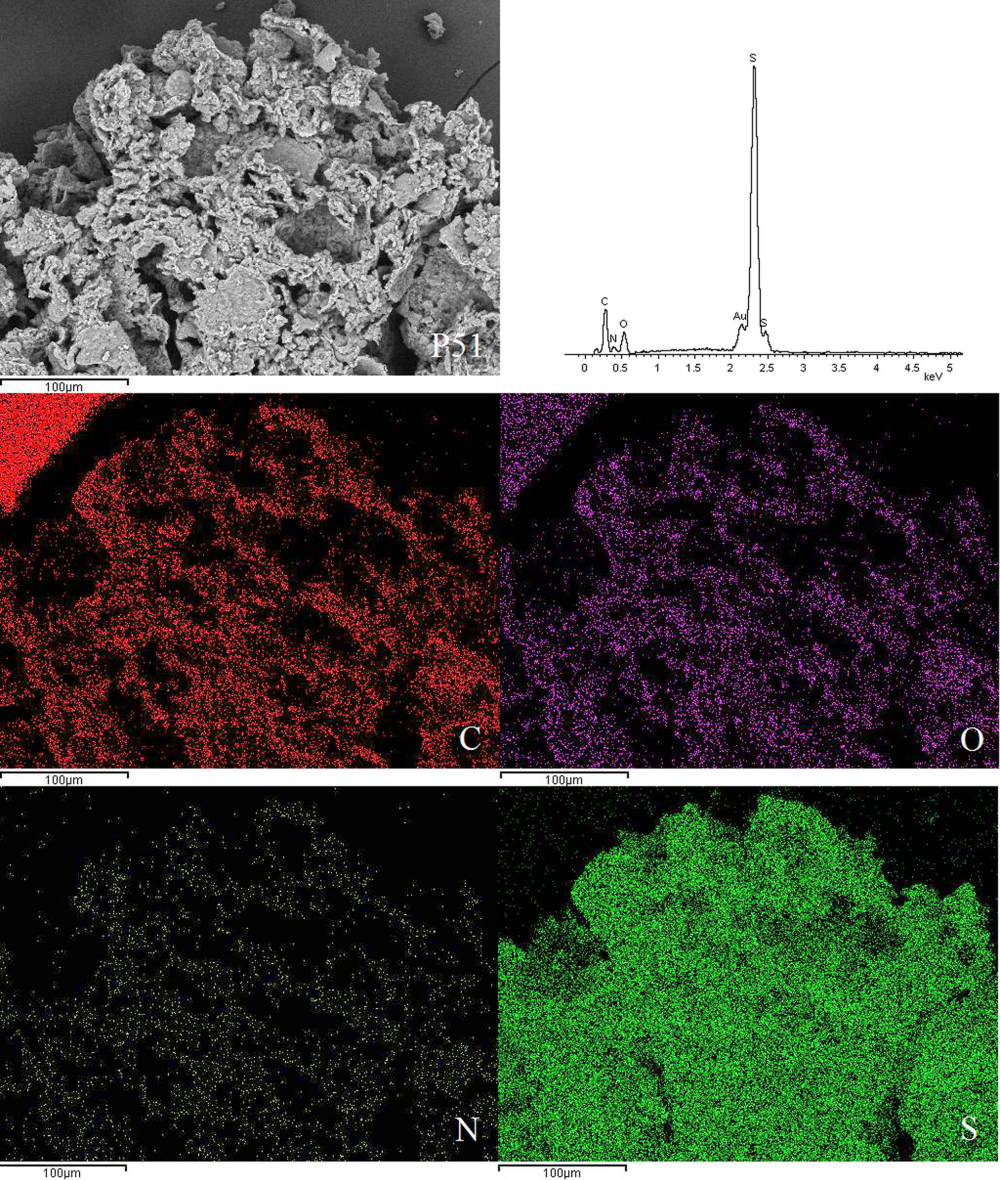
SEM micrographs at ×300, EDX and elemental maps for carbon (C), oxygen (O), sulphur (S), and nitrogen (N) for the same region for polymers P51

EDX spectroscopy of the disulphide polymers showed the existence of elements such as carbon, oxygen, sulphur, nitrogen and these results were further supported by the elemental mapping of the disulphide polymers (Figs. 3, 4, 5, 6, 7, 8). The mapping results demonstrated that all of the disulphide polymers reacted homogeneously due to the similar intensity distribution of the oxygen map and sulphur map. It was found that the intensity distribution of sulphur element in looser polymers (P11, P12, P15, P21, P51) was higher than tighter polymer (P10).

### Chemical reduction of disulphide cross-linked polymers

**Fig. 9:**
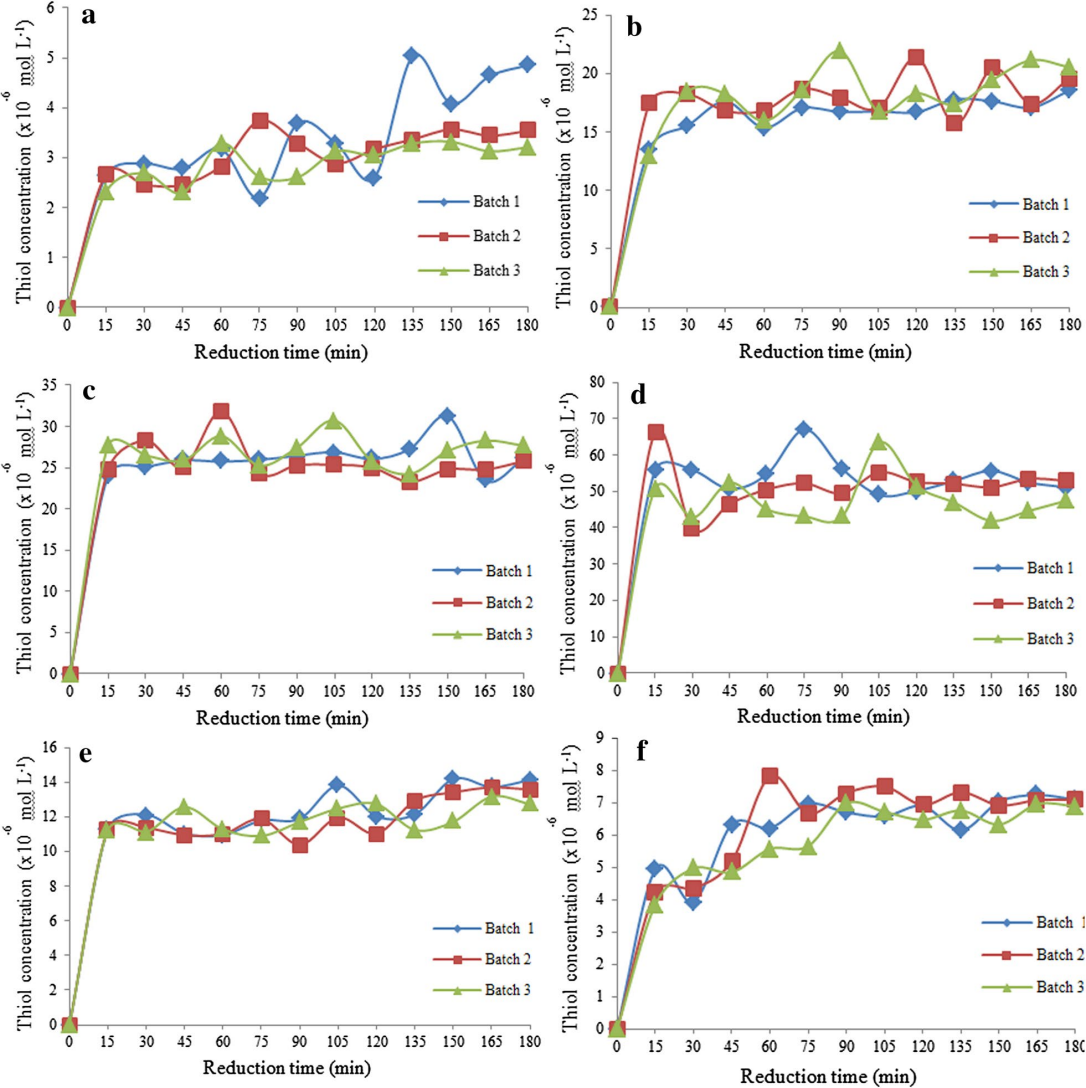
Chemical reduction of polymers **a** P10, **b** P11, **c** P12, **d** P15, **e** P21, and **f** P51

The thiol concentration was highest in the polymer with the highest molar ratio of dithiol monomer (polymer P15) and lowest in the polymer with the lowest molar ratio of dithiol monomer (polymer P10) [5]. The thiol concentration of polymer P15 was approximately 52 × 10^−6^ mol L^−1^. The thiol concentration of polymer P12 was lower (~26 × 10^−6^ mol L^−1^), followed by polymer P11 (~17 × 10^−6^ mol L^−1^), polymer P21 (~13 × 10^−6^ mol L^−1^), polymer P51 (~7 × 10^−6^ mol L^−1^), and polymer P10 (4 × 10^−6^ mol L^−1^) (Fig. 9). Generally, the maximum reduction was achieved after 1 h of reduction time, and the plateau was reached at 3 h of reduction time. Chemical reduction studies showed that all disulphide cross-linked polymers were able to reduced and released free thiol groups.

### In vitro degradation studies

#### Simulated gastric condition

**Fig. 10:**
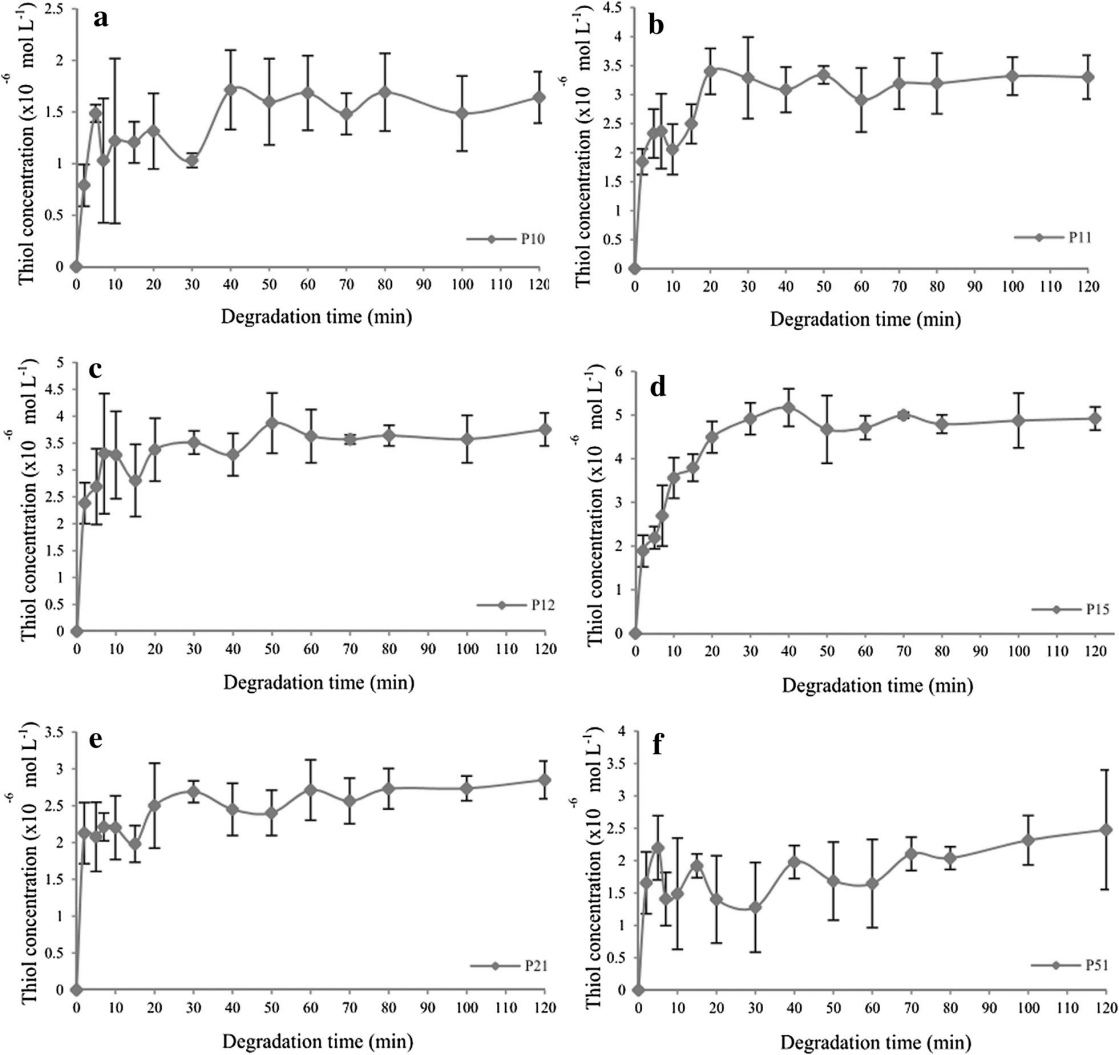
Thiol concentration as a function of dissolution time in the simulated gastric condition over a 120 min period for polymers **a** P10, **b** P11, **c** P12, **d** P15, **e** P21, and **f** P51. Mean ± SD, n = 3

Figure 10 shows the detected thiol concentration for all disulphide polymers in simulated gastric condition.

#### Simulated intestine condition

**Fig. 11:**
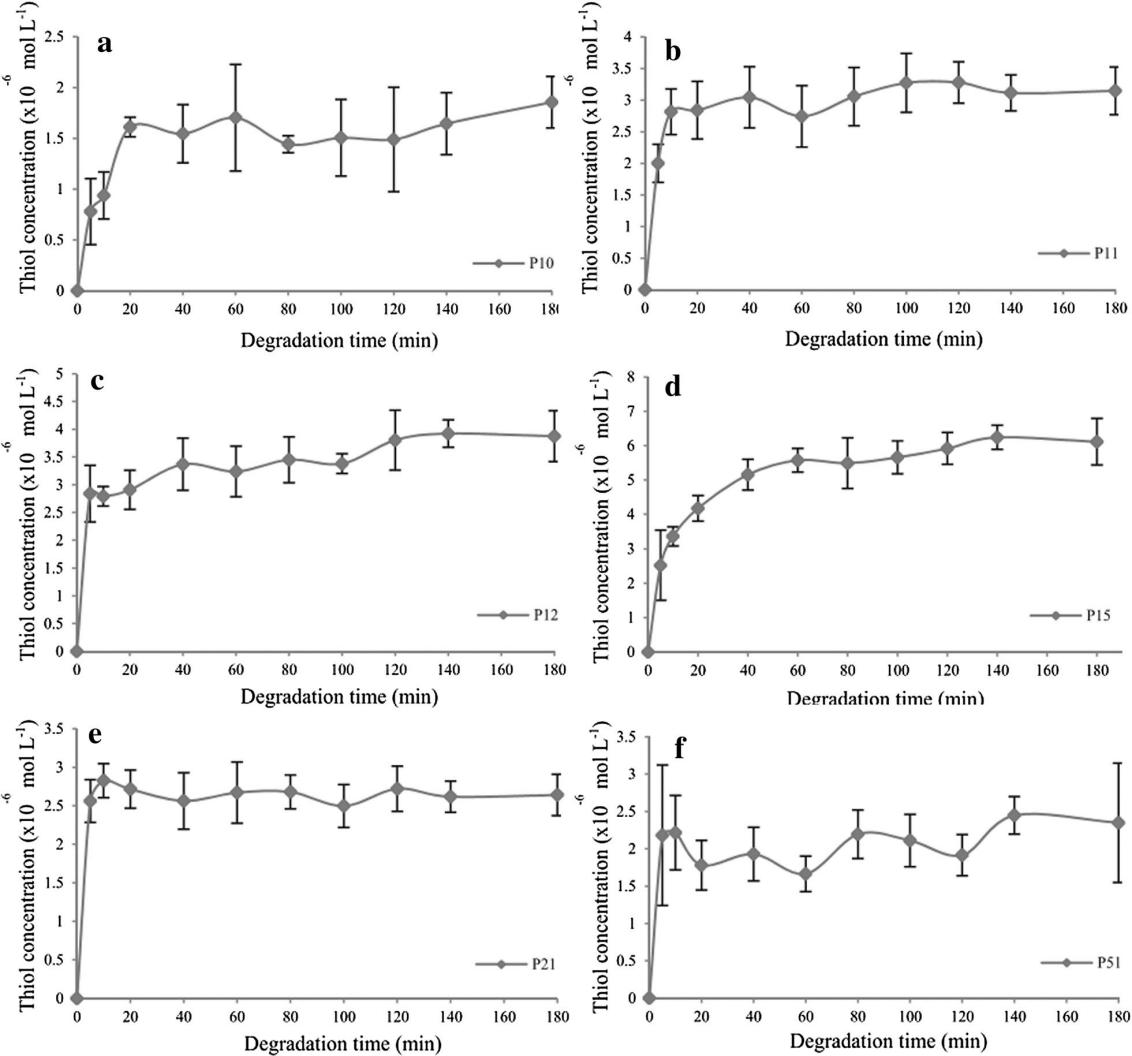
Thiol concentration as a function of dissolution time in the simulated intestine condition over a 180 min period for polymers **a** P10, **b** P11, **c** P12, **d** P15, **e** P21, and **f** P51. Mean ± SD, n = 3

Figure 11 shows the detected thiol concentration for all disulphide polymers in simulated intestine condition.

#### Simulated colon condition

**Fig. 12:**
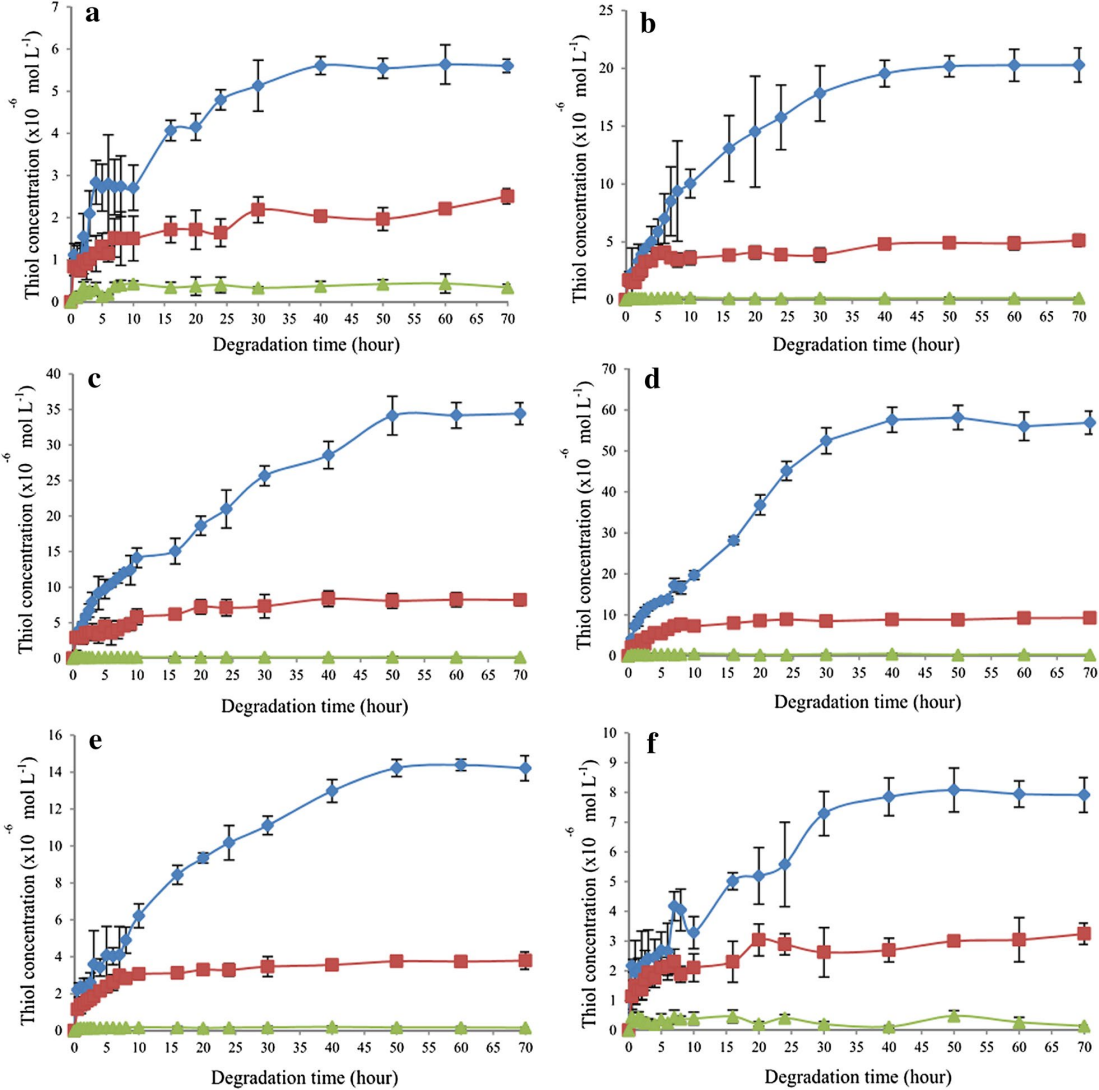
Thiol concentration as a function of dissolution time in simulated colon condition over a 70 h period in polymers **a** P10, **b** P11, **c** P12, **d** P15, **e** P21, and **f** P51. Mean ± SD, n = 3

Figure 12 shows the detected thiol concentration for all disulphide polymers in simulated colon condition.

♦ *Bacteroides fragilis* and polymers; ■ polymers only without bacteria; ▲ bacteria only without polymer

In comparison to the rest of the gastrointestinal tract, the acidic condition of the stomach imposes the greatest threat to the survival of any dosage form that passes through.

### Statistical analysis

Final thiol concentrations of each simulated condition were summarised in Table 3. ANOVA and post hoc Dunnett’s (2-sided) test results showed that the thiol concentrations from the simulated gastric condition were significantly lower (p < 0.05) than those of the simulated colon condition containing the bacteria culture. The thiol concentrations of the disulphide cross-linked polymers in the simulated intestine condition were similar to those in the simulated gastric condition but significantly lower than those in the simulated colon condition with bacteria culture (post hoc Dunnett’s (2-sided) test, p < 0.05) (Table 3). The significantly lower thiol concentration in simulated gastric and intestine condition shows that the polymers degraded minimally in both of the mediums. These results illustrate that the polymers were resistant to the stomach and intestine environments, which is a good feature for a colon drug targeting system.

**Table 3:** Final thiol concentration (×10^−6^ mol L^−1^) of each simulated condition, mean ± SD, n = 3

Incubation medium	P10	P11	P12	P15	P21	P51
Gastric (1)	1.642 ± 0.249	3.302 ± 0.378	3.756 ± 0.308	4.921 ± 0.264	2.851 ± 0.256	2.478 ± 0.923
Intestine (2)	1.856 ± 0.254	3.147 ± 0.377	3.874 ± 0.459	6.113 ± 0.678	2.641 ± 0.269	2.349 ± 0.799
Colon (3)	5.602 ± 0.159	20.288 ± 1.468	34.419 ± 0.541	56.898 ± 2.822	14.211 ± 0.675	7.915 ± 0.585
Statistical analysis	p < 0.05	p < 0.05	p < 0.05	p < 0.05	p < 0.05	p < 0.05
Dunnett (2-sided) (significant)	1 & 3	1 & 3	1 & 3	1 & 3	1 & 3	1 & 3
	2 & 3	2 & 3	2 & 3	2 & 3	2 & 3	2 & 3

In the simulated colon condition, the difference in thiol concentration among the different incubation media was statistically significant (p < 0.05) for all six polymers (Table 4). The thiol concentration in the incubation medium containing the bacteria culture and polymer was significantly higher than that of incubation medium with polymer and bacteria individually [post hoc Dunnett (2-sided) test, p < 0.05] (Table 4). The thiol concentration for the incubation medium with bacteria only was the lowest, and this served as the baseline value.

**Table 4:** Thiol concentrations (×10^−6^ mol L^−1^) of different incubation media at hour 70 in the simulated colon condition

Incubation medium	P10	P11	P12	P15	P21	P51
Bacteria only (1)	0.344 ± 0.076	0.151 ± 0.035	0.157 ± 0.007	0.299 ± 0.101	0.161 ± 0.019	0.146 ± 0.026
Polymer only (2)	2.509 ± 0.179	5.117 ± 0.537	8.212 ± 0.837	9.263 ± 0.151	3.791 ± 0.471	3.244 ± 0.357
Polymer + bacteria (3)	5.602 ± 0.159	20.288 ± 1.468	34.419 ± 0.541	56.898 ± 2.822	14.211 ± 0.675	7.915 ± 0.585
Statistical analysis	p < 0.05	p < 0.05	p < 0.05	p < 0.05	p < 0.05	p < 0.05
Dunnett (2-sided) (significant)	1 & 3	1 & 3	1 & 3	1 & 3	1 & 3	1 & 3
	2 & 3	2 & 3	2 & 3	2 & 3	2 & 3	2 & 3

Mean ± SD, N = 3. The incubation medium containing polymer + bacteria (3) is the control sample

Generally, thiol concentrations of all polymers reached a plateau after incubation for 40–50 h in the presence of *B. fragilis* culture. The ANOVA results showed a significant difference in thiol concentrations among the polymers incubated with bacteria culture. The thiol concentration was highest for polymer P15, followed by P12, P11, P21, P51, and P10.

Polymer P15 had the highest thiol concentration among the six polymers tested when incubated in the simulated colon condition in the presence of *B. fragilis* culture. These results are in agreement with those reported by Lim et al. [5], who found that the polymer with the molar ratio of 1:5 (trithiol monomer:dithiol monomer) had the highest thiol concentration in the simulated colon condition. Theoretically, polymer P15 had the loosest polymer network among the six polymer formulations tested. This feature allowed the polymer to expand in solution, thus allowing access of solvent into the polymeric network [9]. In contrast, polymers P10, P11, P12, P21, and P51 had a confined polymeric network and a lower rate of expansion in solution. Bacterial reduction was more favoured in loose polymers compared to confined polymers.

## Conclusions

In conclusion, a novel branch-chained disulphide cross-linked polymer P15 was successfully synthesised using the oxidation polymerisation method. The synthesised polymer was able to withstand the harsh environment of the simulated gastric and intestine conditions and was reducible in the simulated colon condition containing *B. fragilis* culture. Therefore, polymer P15 has potential for use as a colon specific drug delivery system. However, much work is needed to develop dosage forms for more effective delivery of drugs to the colonic region to establish their stability and feasibility for use in a pharmaceutical dosage form and to achieve optimum treatment efficacy for various colon diseases.

## Additional file

**Additional file 1.** Additional figures.

## Abbreviations

GI: gastrointestinal
TFA: trifluoroacetic acid
DCM: dichloromethane
HOBt: hydroxybenzotriazole hydrate
EDC: N-(3-dimethylaminopropyl)-N′-ethylcarbodiimide
TLC: thin layer chromatography
TES: triethylsilane
DMSO: dimethyl sulphoxide
FT-IR: Fourier transform infrared spectroscopy
^1^H-NMR: proton nuclear magnetic resonance spectoscopy
CDCl_3_: deuterated chloroform
CHNS: elemental analysis
SEM-EDX: scanning electron microscope-energy dispersive x-ray
HPLC: high performance liquid chromatography
EDTA: ethylenediaminetetraacetic acid
UV: ultraviolet
ANOVA: one-way analysis of variance
HSD: honest significant difference
