# Supplementary material for: Colon targeted drug delivery of branch-chained disulphide cross-linked polymers: design, synthesis, and characterisation studies
Source: Chem Cent J. 2016 Nov 29;10:77. doi: 10.1186/s13065-016-0226-4 (PMC5129663; doi:10.1186/s13065-016-0226-4)
Supplement: Supplementary file 1 — Additional file 1. Additional figures. [file 13065_2016_226_MOESM1_ESM.docx]

**
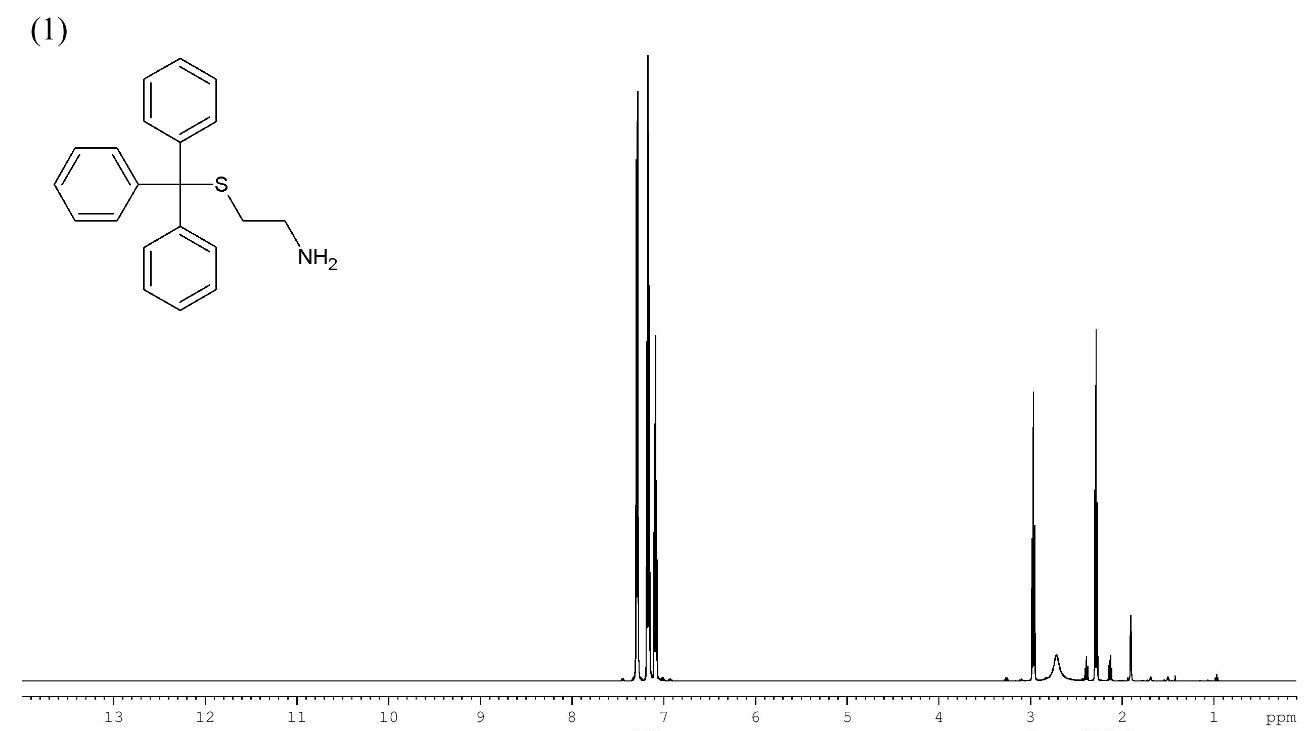
Supplementary information**

**Figure S1. ^1^H-NMR spectrum of compound 1**


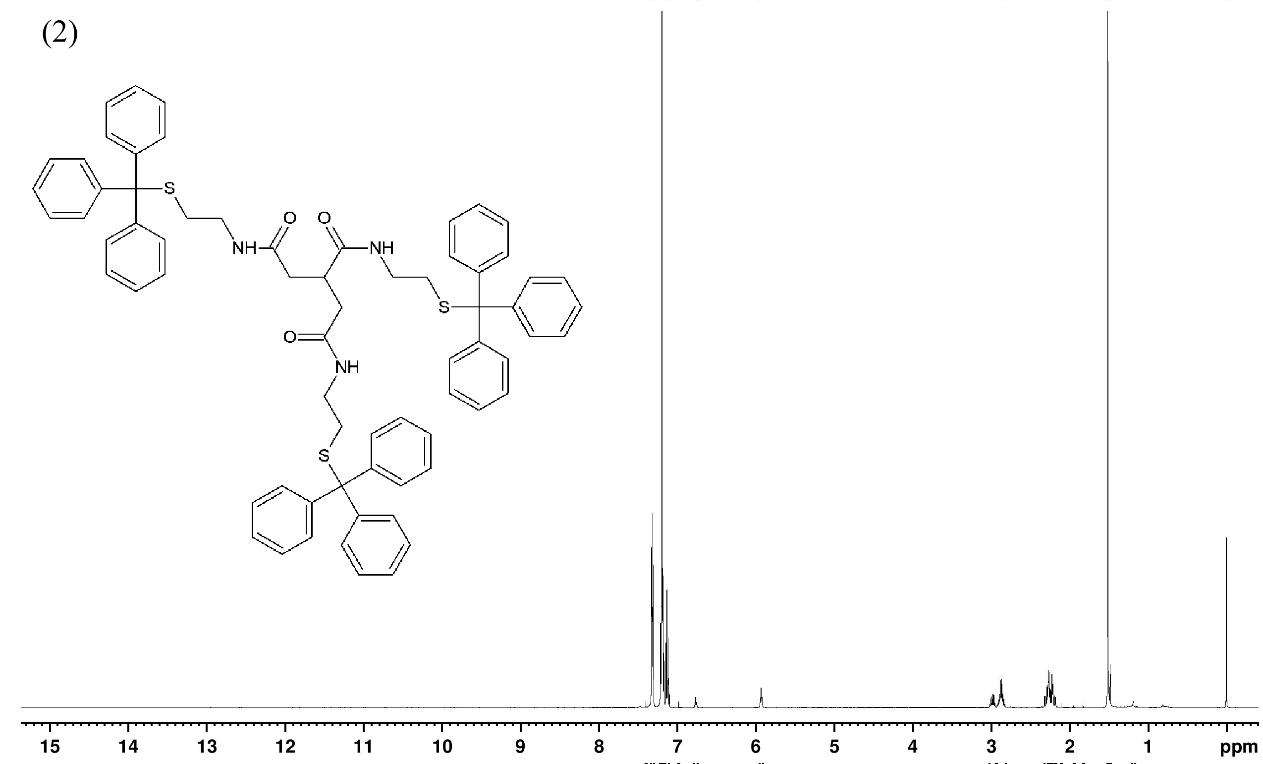


**Figure S2. ^1^H-NMR spectrum of compound 2**


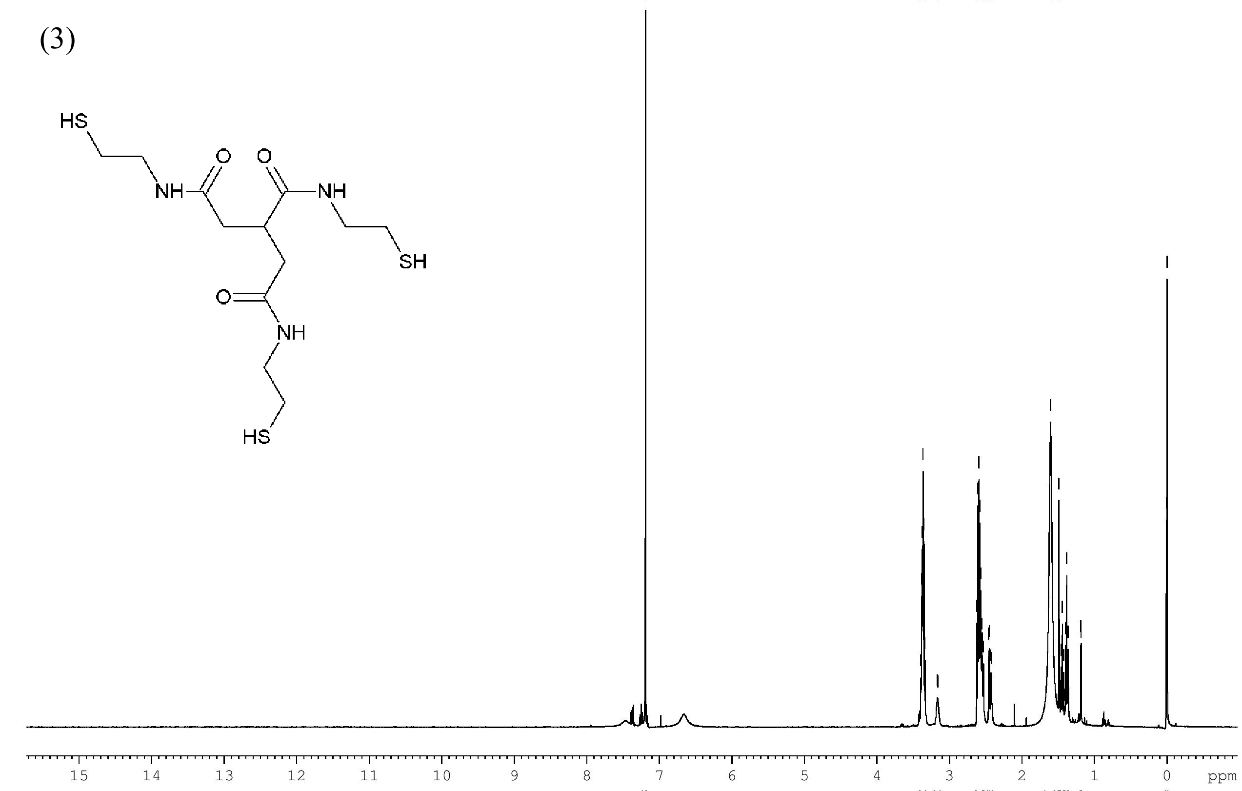


**Figure S3. ^1^H-NMR spectrum of compound 3**
